# Assessment of Changes in the Fat Profile of House Cricket Flour during 12 Months of Storage in Various Conditions

**DOI:** 10.3390/foods13162566

**Published:** 2024-08-16

**Authors:** Agnieszka Orkusz, Lucyna Dymińska, Anna Prescha

**Affiliations:** 1 Department of Biotechnology and Food Analysis, Wroclaw University of Economics and Business, 53-345 Wroclaw, Poland; 2 Department of Bioorganic Chemistry, Wroclaw University of Economics and Business, 53-345 Wroclaw, Poland; lucyna.dyminska@ue.wroc.pl; 3 Department of Dietetics and Bromatology, Wroclaw Medical University, 50-556 Wroclaw, Poland; anna.prescha@umw.edu.pl

**Keywords:** edible insects, temperature, storage, fatty acids, chemical composition, dietary indicators, oxidative stability

## Abstract

Considering *Acheta domecticus* flour’s growing importance and utilization as an ingredient in many food products, research on its storage is essential. The objective of this study was to determine the chemical and nutritional fat profile of house cricket (*Acheta domesticus*) flour during storage for 12 months under different storage temperatures (−18 °C, +4 °C, and +20 °C in two variants, with and without access to light). Insect flour was studied using Fourier-transform infrared spectroscopy (FTIR). The fatty acids content was determined, and dietary indicators were calculated. The acid value, peroxide value, and anisidine value were also determined, and differential scanning calorimetry was performed. The results obtained from spectroscopic analysis of *Acheta domesticus* flour were consistent with the biochemical data. During the 12-month period of flour storage, the storage temperature significantly influenced the percentage composition of identified groups of fatty acids and the values of all presented ratios and dietary indices. During storage at refrigerated temperatures (−18 °C and +4 °C), no changes were observed in the fatty acid content and dietary indices, indicating that refrigerated temperatures provide oxidative stability to flour during 12 months of storage. Samples stored at 20 °C had higher acid values (AV), peroxide values (PV), and anisidine values (p-AV) compared to samples stored at lower temperatures (4 °C and −18 °C). Simultaneously, an increase in SFA and MUFA, as well as a decrease in PUFA and UFA, was noted in samples stored at room temperature. Storing cricket flour at lower temperatures when the storage period will be more than 12 months is essential to restrict the occurrence of fat oxidation. Elevated temperatures and exposure to light have a notable effect in hastening oxidation mechanisms, reducing thermal resilience, and inducing more pronounced alterations in the quality of fats.

## 1. Introduction

Edible insects are well-known for their high nutrient content, including protein, fat, essential amino acids, polyunsaturated fatty acids, minerals (Fe, Ca, Zn), and vitamins (A, B_6_, B_12_) [1]. The growing interest in using insects as a nutritional source stems from two main concerns. Primarily, they have the potential to address malnutrition, particularly in developing countries. Secondly, they are recognized as an environmentally friendly protein alternative to meet global food requirements [2]. 

The land required (in square meters) per gram of protein production in livestock versus insect farming is estimated at 254 for cows, 63 for pigs, 51 for chicken, and 18 for insects. The amount of water (in liters) needed per gram of protein production is 112 for beef, 57 for pork, 34 for chickens, and 2 for crickets. In addition, the fossil energy input (in megajoules) per kilogram of protein output is 385 for beef, 340 for chicken, and 120 for crickets [3].

In 2021, the market value of edible insects was estimated to be 3.2 million U.S. dollars, and the market value of edible insects in the Asia-Pacific region, Latin America, Europe, North America, the Middle East, and Africa would reach, respectively, 476.9 million USD, 290.6 million USD, 261.5 million USD, 153.9 million USD, and 38.7 million USD by 2023. These estimated values are significantly higher than the market values of insects in 2018, which amounted to, respectively, 173.9 million USD for the Asia-Pacific region, 92.2 million USD for Latin America, 82.1 million USD for Europe, 44.1 million USD for North America, and 14.2 million USD for the Middle East and Africa. Consequently, insects could present numerous opportunities for the food industry and offer consumers readily available alternative protein sources [3]. 

Worldwide, the following numbers of edible insect species are recorded: 659 beetles, 362 caterpillars, 321 ants, bees, and wasps, 278 grasshoppers and locusts, 237 true bugs, 61 dragonflies, 59 termites, 37 cockroaches and flies, and 45 others [3]. Many nations and ethnic groups, especially in Australia, South America, Asia, and Africa, have an old tradition of eating insects [4,5,6,7,8]. In Western culture, insects are not commonly perceived as food [9,10,11]. Their consumption often elicits disgust and rejection [12,13]. However, if insects are introduced into other food products in powdered form, it is easier to persuade consumers to try them, especially if these are commonly consumed and liked products, such as bread, cakes, and pasta [14,15]. 

Recently, many studies have focused on designing products containing insects (mainly *Acheta domesticus*) and analyzing their properties. The most commonly enriched products include bread [16,17,18,19,20,21,22,23], pasta [24,25,26,27,28,29], pancakes [30,31], snacks [32,33], biscuits [34,35,36,37,38], and meat products [39,40,41].

Considering cricket flour’s growing importance and utilization as an ingredient in many food products, research on its storage is essential. So far, storage studies have yet to be the subject of scientific research. Therefore, it is essential to undertake such research to determine the optimal storage conditions to guarantee the appropriate quality of cricket flour, characterized by unchanged taste and aroma.

The stability of flour and its properties are influenced by temperature, humidity, packaging material, and storage time [40,41,42,43,44,45,46,47,48,49,50]. Wheat and rice flour can be stored for 12 to 16 months [51,52,53], while, e.g., potato and amaranth flour can be stored for about 6 months [54,55]. Flour is stored mainly in paper and plastic packaging made of polyethylene and polypropylene [56,57,58]. Plastic packaging provides better protection against moisture and microbial growth, leading to a longer flour shelf life while maintaining appropriate quality.

The loss of quality in wheat flour is mainly due to lipid oxidation [44]. Cricket flour, rich in fat and unsaturated fatty acids [59], is particularly susceptible to oxidation [60]. However, despite abundant research on insects, few studies focus on lipid oxidation during long-term storage [60,61,62].

Kamau et al., 2018 [60] investigated the oxidative stability of house cricket meals under different storage conditions and in different packaging materials. The samples were boiled, solar-dried, milled, and packaged into polypropylene (PP), plastic (PL), and polyethylene (PL) packages. The samples were then stored in refrigerated (4 C) and ambient conditions for six months. The study employed peroxide value (PV) and p-anisidine value (P-AV) as indicators of oxidation stability. An increase in PV and P-AV during storage indicated lipid oxidation and the formation of both primary and secondary oxidation products depending on the storage environment. The PV and the P-AV values showed a considerable rise under ambient and refrigerated conditions, indicating ongoing lipid oxidation. Refrigerated storage helped maintain the stability of fatty acids in the adult house cricket meal by slowing down oxidation processes, thereby preserving the quality of the product over time. The research highlighted that oxidation rates were directly related to storage temperature, with higher oxidation occurring under ambient conditions than refrigeration. 

Singh et al., 2020 [61] investigated seven different killing methods for the house cricket (blanching, steaming, freezing, carbon dioxide injection, vacuum, plastic bags, and a combined method: carbon dioxide + blanching) on the oxidative stability of cricket powder during a 16-week storage period. As the authors expected, high-temperature thermal treatments produced the highest lipid oxidation, which increased with storage time. The authors emphasized that the killing method may not be a critical factor influencing the oxidation stability of cricket powder, possibly due to its inherent radical scavenging capacity.

Marzoli et al., 2023 [62] focused on the oxidation stability of *Acheta domesticus* powder produced using three different processes (medium-temperature drying (80 °C), high-temperature drying (120 °C), and lyophilization) during a one-year storage period at room temperature. This study monitored changes in peroxide values (PVs) and aldehyde contents to evaluate lipid oxidation. PVs and aldehydes increased during storage. The study found that different drying methods influenced oxidation levels, with oven-dried samples exhibiting higher peroxide values and aldehyde contents than lyophilized samples. However, the authors did not specify what “room temperature” means, which is a significant omission, as temperature significantly affects lipid changes.

There is a clear need for comprehensive research to better understand the long-term effects of storage on the oxidative stability of cricket flour. That is why this study aimed to determine the chemical and nutritional fat profile of house cricket flour (*Acheta domesticus*) stored for 12 months at different temperatures. 

This study aimed to determine the acid value (AV), peroxide value (PV), and anisidine value (p-AV) and to perform differential scanning calorimetry (DSC). Additionally, the fatty acid profile and FTIR/ATR spectra were analyzed to allow for a more precise assessment of the fat composition and its changes under different storage conditions. Furthermore, the atherogenic index (AI), thrombogenic index (TI), and hypocholesterolemic/hypercholesterolemic ratio (h/H) were calculated based on the fatty acid profile. These indices are essential for evaluating the quality of dietary fats and their impact on cardiovascular risk. Higher AI and TI values are linked to an increased risk of cardiovascular and metabolic diseases, whereas a higher h/H ratio offers protective benefits [63,64].

## 2. Materials and Methods

### 2.1. Research Material

**Figure 1:**
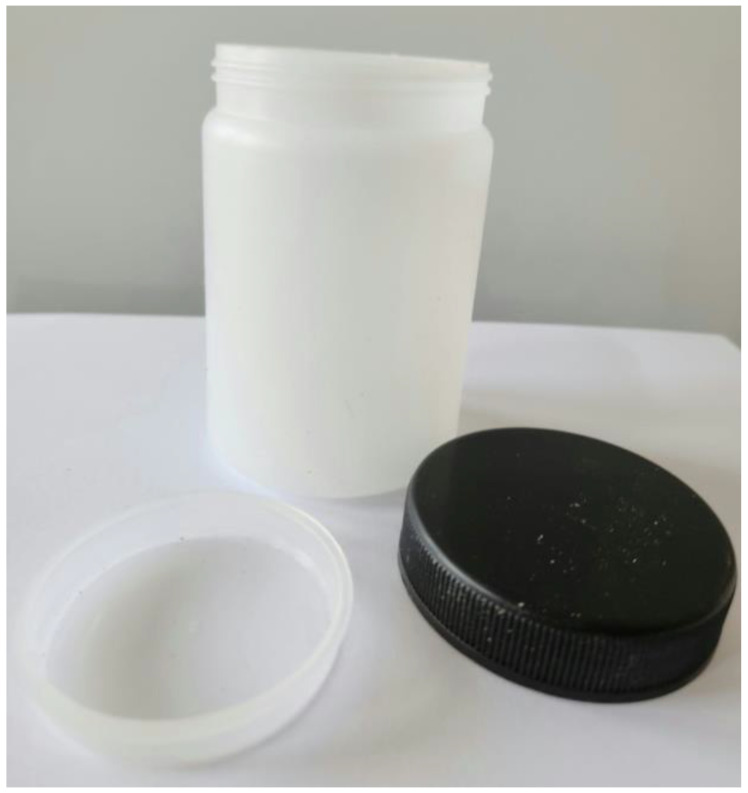
Plastic container (HDPE) with a capacity of 250 mL used for storing flour.

The research material was *Acheta domesticus* flour (2 kg) purchased commercially in metalized paper packaging. The flour was divided into 12 plastic containers in 150 g portions. Tight containers made of high-density polyethylene (HDPE) with a push-in cork and cap with a capacity of 250 mL (Figure 1), recommended for storing granules and powders, were used. The containers have a wide neck, enabling convenient container filling.

The samples were stored for 12 months at controlled temperatures (at room temperature either +20 °C in two variants, with and without access to light, or in cold storage rooms at + 4 °C and −18 °C) (Table 1). 

Temperature and humidity were measured using an electronic data logger with a probe on a cable and an LCD, allowing direct data readings from the device. Four devices were used in the study, with probes placed in the refrigerator, freezer, laboratory cabinet, and outside laboratory cabinet. The loggers continuously measured both temperature and humidity. Temperature and humidity data were regularly monitored throughout the entire 12-month storage period.

The determinations were made in the control sample immediately after purchase (storage time “0”) and in the flours (stored in four different conditions) after 12 months of storage. 

### 2.2. Infrared Measurements

FTIR/ATR spectra were obtained in the 4000–400 cm^−1^ range with a Nicolet 6700 spectrometer (Thermo Fisher Scientific, Waltham, MA, USA) and a portable ATR set. The resolution of these measurements was set to 2 cm^−1^. Before analysis, all FTIR spectra were uniformly processed. In the first step, the spectra were compared and analyzed using commercial computer software (OriginPro 2024, OriginLab Corp., Northampton, MA, USA). This analysis included background subtraction and deconvolution of the experimental bands into the Lorentz components [65]. 

### 2.3. Fat Determination and Gas Chromatographic Analysis

The fat concentration was assessed according to the AOAC Official Method 991.36 (AOAC, 1990) [66]. Briefly, 3 g of a sample was weighed into a cellulose thimble and mixed with extractable-free sand before being placed in a Soxhlet extraction apparatus. Fat extraction was performed for 30 min with petroleum ether in a pre-weighed round-bottomed flask at a condensation rate of 5 drops/minute. After the evaporation of the solvent, the flask was dried and weighed, and the fat concentration in the sample was calculated. For the analysis of fatty acid (FA) profiles, the lipid samples underwent conversion into their corresponding methyl esters using the AOCS Official Method Ce 2-66 [67]. Subsequently, the FA methyl esters were analyzed via gas chromatography utilizing a Perkin Elmer Clarus 580 gas chromatograph (PerkinElmer, Waltham, MA, USA) equipped with a 105-m silica capillary column containing an Rtx2330 stationary phase (Restek Corporation, Bellefonte, PA, USA) and a flame-ionization detector (GC-FID) (PerkinElmer, Waltham, MA, USA). Hydrogen served as the carrier gas at a constant flow of 1.5 mL/min. The detector and injector were maintained at a temperature of 240 °C. The column temperature was initially set at 165 °C for 10 min, then increased to 220 °C at 2 °C/min. Fatty acids were represented as a percentage relative to the total amount of methyl esters. The values for saturated fatty acids, monounsaturated fatty acids, and polyunsaturated fatty acids were presented as weight percentages, representing the proportion of these fatty acids in the total fatty acid content.

### 2.4. Dietary Indicators

The atherogenic index (AI), thrombogenic index (TI) [68], and hypocholesterolemic/hypercholesterolemic ratio (h/H) [69,70] were determined using the following equations:

AI = (C12:0 + 4 × C14:0 + C16:0)/(MUFA + n − 6 + n − 3);

TI = (C14:0 + C16:0 + C18:0)/(0.5 × MUFA+ 0.5 × n − 6 + 3 × n − 3 + n − 3/n − 6);

h/H = (C18:1 *c*9 + C18:2 n − 6 + C18:3 n − 3 + C20:3 n − 6 + C20:4 n − 6 + C20:5 n − 3 + C22:5 n − 3)/(C12:0 + C14:0 + C16:0).

where MUFA is the acronym for “monounsaturated fatty acids”.

### 2.5. Oxidative Stability

The acid, peroxide, and p-anisidine values were determined using fat extracted from cricket flour in accordance with CEN ISO 660:2009 [71], CEN ISO 3960:2010 [72], and CEN ISO 6885:2008 [73], respectively. The accelerated oxidation tests of cricket flour were performed using differential scanning calorimetry (DSC) at a constant heating rate of 10 °C/min. Testing was performed using a Perkin Elmer differential scanning calorimeter (Pyris 6) (PerkinElmer, Waltham, MA, USA), following the procedure detailed by Grajzer et al., 2015 [74].

### 2.6. Statistical Analysis

The data underwent statistical processing [75] by calculating arithmetic means and standard deviations. To assess the effect of different storage conditions on the lipid profile of insect flour, PCA was performed on the integral intensities of selected FTIR bands. All analyses were conducted in triplicate. One-way analysis of variance (ANOVA) was employed to assess variances among the examined parameters. The mean values were further examined using Duncan’s multiple-range test after detecting a significant effect. Differences with a *p*-value < 0.05 were considered statistically significant.

### 2.7. Ethical Statement

This study did not require ethical review as it was based on a commercially available food product. 

## 3. Results and Discussion

This study utilized analytical methods to comprehensively assess the quality and stability of fats in cricket flour under various storage conditions. The authors examined FTIR/ATR spectra and fatty acid profiles to understand the fat composition of the flour and its alterations under different storage conditions. Moreover, the study calculated the atherogenic index, thrombogenic index, and hypocholesterolemic/hypercholesterolemic ratio based on the fatty acid profile. The degree of decomposition of the fat extracted from the cricket flour was assessed by commonly used quality parameters, including acid value (AV), peroxide value (PV), and *p-*anisidine value (*p-*AV). Each method provides specific information on fat oxidation and stability aspects, allowing for a comprehensive understanding of the changes occurring in stored fats. The acid value measures the amount of free fatty acids, which indicates hydrolytic and oxidative degradation of fats. The peroxide value measures primary oxidation products such as peroxides. The anisidine value assesses secondary oxidation products, including aldehydes. In addition, the susceptibility of the cricket flour to oxidation was assessed in accelerated oxidation conditions at temperatures ranging from 50 to 350 °C in an oxygen atmosphere using the DSC method. This test allowed us to monitor the oxidation process by determining the temperature during the oxidation’s induction, propagation, and termination phases.

**Figure 2:**
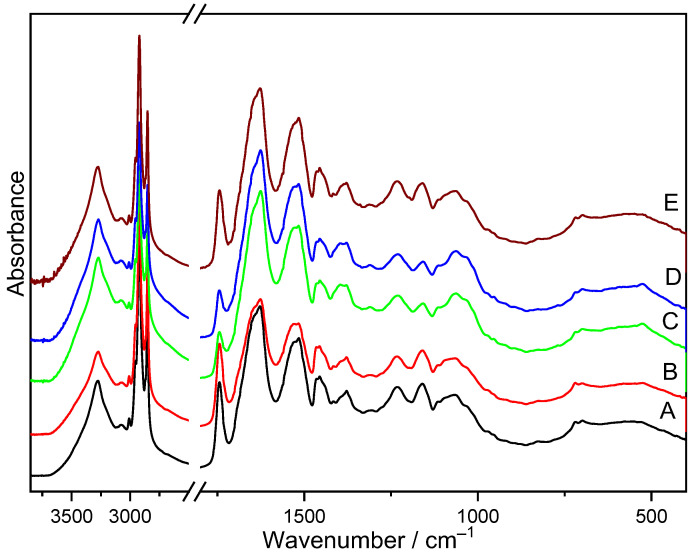
FTIR spectra of the studied samples: A (Control sample), B (4 °C), C (20 °C), D (20 °C + light), and E (−18 °C).

Infrared analysis was performed to determine the effect of storage conditions on the content of main polymers in insect cells. FTIR spectra of the analyzed samples were recorded in the range of 4000 to 400 cm^−1^ (Figure 2). 

**Figure 3:**
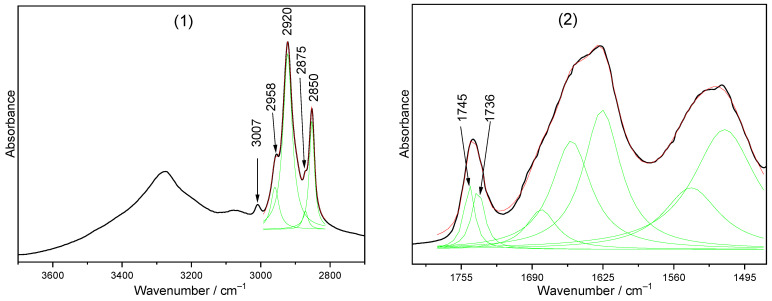

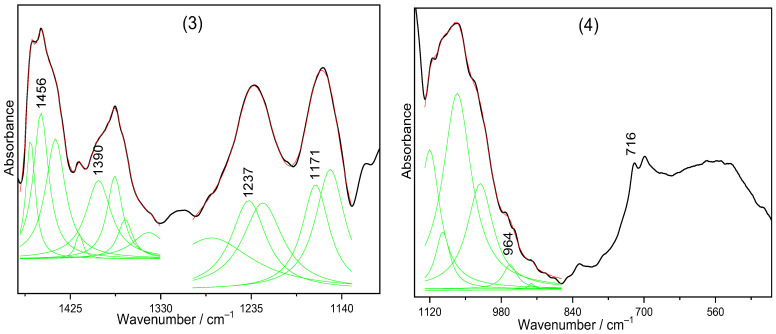
FTIR spectra for the control sample (storage time “0”) in the ranges: 3700–2700 cm^−1^ (**1**), 1800–1475 cm^−1^ (**2**), 1480–1100 cm^−1^ (**3**), and 1150–450 cm^−1^ (**4**).

Figure 3 shows the analyzed bands and Lorentzian components for which integral intensities were determined.

All of the integral intensities of the observed bands were standardized with the statistical R^2^ determination coefficient. For this purpose, several simulations of Lorentz deconvolution were performed using a wide and variable number of components. The best fit between the experimental and theoretical spectral contour was achieved when their statistical values’ R^2^ ranged between 0.98 and 1.0 and the convergence coefficient χ^2^ was 10^−6^.

**Figure 4:**
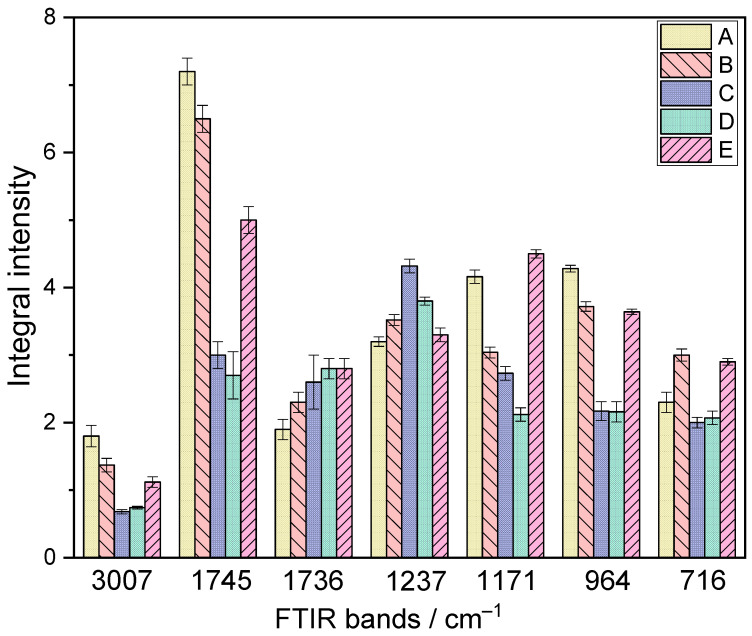
Differences in the integral intensities of the bands at 3007, 1745, 1736, 1237, 1171, 964, and 716 cm^−1^ for samples: A (control sample), B (4 °C), C (20 °C), D (20 °C + light), and E (−18 °C).

Changes in fat content were determined by the analysis of differences in integral intensities (I) of bands at 3007 cm^−1^, 1745 cm^−1^, 1736 cm^−1^, 1237 cm^−1^, 1171 cm^−1^, 964 cm^−1^, and 716 cm^−1^ (Figure 4) [65,76,77,78,79,80]. 

Useful information can be obtained by comparing the integral intensities (I) of the bands observed at about 3007 cm^−1^ and 964 cm^−1^, which correspond to ν_as_(=C-H) and δ(C-C=C) vibrations, respectively. The intensities of these bands fulfill the relation: IA > IE ≥ IB > IC ≈ ID. These data show that the amount of unsaturated fatty acids decreases during storage. The most significant decrease is noticeable in samples stored at 20 °C (samples C and D). This suggests higher levels of saturated fatty acids in the stored samples. However, the fatty acid chains are broken in samples C and D. This is evidenced by lower integral intensities of the bands at the wavenumber 716 cm^−1^. This band is characteristic of vibration γ(C-C-C). 

FTIR spectra measured for the studied samples were also analyzed using the Principal Component Analysis (PCA) procedure to visualize the spectroscopic data’s main trends (Table 2).

The Principal Component Analysis results showed statistically significant differences among the samples stored under different conditions. Principal Component 1 (PC1) explained 83.46% of the variance for the integral intensities of the band at 3007 cm^−1^, while Principal Component 2 (PC2) explained 16.38%. For the band at 964 cm^−1^, PC1 and PC2 explained 83.26% and 16.40% of the variance, respectively, and for the band at 716 cm^−1^, they explained 83.32% and 16.40%, respectively. Together, these two components represented 99.84%, 99.66%, and 99.72% of the total variance for the respective bands, indicating that a significant explanation of the data variability can be constructed based on these two principal components.

The FTIR bands in the 1775–1725 cm^−1^ range may be helpful for the identification of the changes in the carboxylic group. The component at about 1745 cm^−1^ corresponds to the ν(C=O) vibrations of the ester groups. The integral intensity of this band fulfills the relation: IA > IB > IE > IC > ID, showing that samples A and B exhibit a greater content of the ester group in fats. When stored at 20 °C, fat decomposes into free fatty acids and glycerol. The band at 1736 cm^−1^ corresponds to the stretching vibration of the C=O bonds in free carboxyl groups, aldehydes, and ketones. The intensities of this band are higher for stored samples, suggesting the formation of new chemical components. PC1 accounted for 82.60% of the variance and PC2 for 16.41% of the band integral intensities at 1745 cm^−1^, while for the band at 1736 cm^−1^, PC1 accounted for 83.45% and PC2 for 16.39% of the variance (Table 2).

The band at 1171 cm^−1^ corresponds to ν(C−O−C) and ν(C−OH) vibrations. Comparing the integral intensities of this band for A, B, C, and D samples reveals the same relationships as in the 1745 cm^−1^ band. This confirms the previous conclusion that the number of ester bonds decreases during sample storage. The integral intensity of this band for sample E is high compared to the other samples. This is due to the creation of new chemical components with C-O-C and C-OH connections during storage. PC1 for the integral intensities of the band at 1171 cm^−1^ was 83.25%, while PC2 was 16.40% (Table 2).

Integral intensities of the Lorentzian component at 1237 cm^−1^ increase for samples B, C, D, and E compared with the control sample (A), suggesting the increase of hydrogen bond number in storage samples. The integral intensity of this band fulfills the relation: IA ≤ IE < IB < ID < IC. This is possible because new chemical compounds are formed in the studied samples, between which hydrogen interactions may occur. For this data, PC1 was 83.41%, and PC2 was 16.40% (Table 2). 

The Principal Component Analysis results showed that the first principal component accounted for 82.60% to 83.46% of the total variance, while the second principal component explained an additional 16.38% to 16.41% (Table 2). These two components represented 99.01% to 99.84% of the total variance, indicating that a significant explanation of the data variability can be constructed based on these two principal components. The high percentage of the variance explained by PC1 in the bands associated with unsaturated fatty acids (3007 cm^−1^ and 964 cm^−1^) and saturated fatty acids (716 cm^−1^) indicates significant differences in fat stability resulting from storage conditions. These results suggest that storage at 20 °C, especially with light exposure, leads to the degradation of unsaturated fatty acids and the breakdown of fatty acid chains.

**Figure 5:**
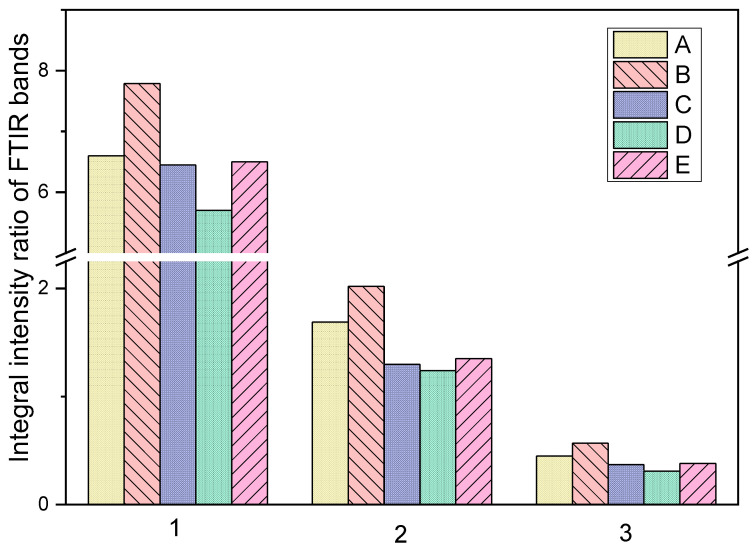
Differences in the integral intensity ratios of the bands at 2920 and 2958 cm^−1^ (1), 2850 and 2875 cm^−1^ (2), 1456 and 1390 cm^−1^ (3). A (control sample), B (4 °C), C (20 °C), D (20 °C + light), and E (−18 °C).

The bands corresponding to the stretching vibrations (ν_s_—symmetric, ν_as_—asymmetric) and deformation vibrations (δ) of methyl and methylene groups can be used as analytical bands to compare the content of lipids and fatty acids in the studied samples [81,82]. These bands are observed at the wavenumbers: 2958—ν_as_(CH_3_), 2920—ν_as_(CH_2_), 2875—ν_s_(CH_3_), 2850—ν_s_(CH_2_), 1456—δ(CH_2_), and 1390 cm^−1^—δ(CH_3_). The integral intensity ratios of the bands at 2920 and 2958, 2850 and 2875, and 1456 and 1390 cm^−1^ were calculated (Figure 5). For sample B, the number of saturated bonds -C-C—increases, which increases the CH_2_/CH_3_ ratio. For samples C and D, the fatty acid chains are broken and the number of methyl groups increases, which causes the CH_2_/CH_3_ ratio to decrease compared to the control sample (A). 

The results obtained from spectroscopic analyses of the *Acheta domesticus* flour were consistent with the biochemical data.

In the fatty acid profile of insect flour (control sample), fatty acids ranging from C 14:0 to C 18:3 n-3 were identified (Table 3). It was demonstrated that unsaturated fatty acids (UFA—Unsaturated Fatty Acids) predominated. Their content totaled 63.82%, including monounsaturated fatty acids (MUFA), which accounted for 28.66%, and polyunsaturated fatty acids, which accounted for 35.16%. Saturated fatty acids accounted for 36.22% of the total content of all acids. Among saturated fatty acids, palmitic acid was predominant (25.57%), oleic acid (27.63%) predominated among the monounsaturated fats, and linoleic acid (33.27%) predominated among the polyunsaturated fats (Table 3). The determined fatty acid composition of *Acheta domesticus* flour is consistent with other authors’ research [83].

It was found that storage conditions influenced the content of fatty acids in insect flour. It has been demonstrated that in samples stored at refrigerated temperatures for 12 months, the fatty acid profile remained unchanged compared to the control sample. For flours stored at temperatures of −18 °C and +4 °C, no significant differences were observed in SFA, MUFA, PUFA, UFA, and PUFA/SFA content. Therefore, storing house cricket flour for 12 months under refrigerated conditions, both at −18 °C and +4 °C, ensured the oxidative stability of the fat, protecting the flour from oxidation and thus keeping the fatty acid profile of the samples unchanged. 

It was noted that storing samples for 12 months at room temperature, both with and without access to light, promoted the oxidation of the fat contained in the flour. As a result, changes in the fatty acid profile were observed. Particularly unfavorable from a nutritional standpoint was the decrease in the percentage of PUFA (including linoleic and linolenic acid) and the increase in SFA content compared to the control sample. This relationship contradicts current scientific recommendations, which advocate for minimizing the intake of saturated fatty acids in the diet and simultaneously recommend shifting consumption from saturated fatty acids to polyunsaturated fatty acids [84].

After 12 months of storage, the percentage content of SFA increased by 11.78% and 17.17% in samples without and with access to light, respectively. The increase in saturated fatty acid content was mainly due to the rise in palmitic acid (C 16:0) and stearic acid (C 18:0). Storing samples at 20 °C without and with access to light resulted in a decrease in the overall content of polyunsaturated fatty acids by 12.88% and 20.82%, respectively. This decrease stemmed from a reduction in the content of linoleic acid (C 18:2 n-6) and linolenic acid (C 18:3 n-3) by 12.50% and 24.56% (for samples without access to light) and 22.06% and 25.43% (for samples stored at room temperature with access to light). The decrease in the percentage share of polyunsaturated fatty acids can be considered a reduction in the nutritional value of the flour’s fat.

Unsaturated fatty acids are particularly susceptible to degradation, with oxidation being one of the primary mechanisms of it. The degree of oxidation tends to increase with the number of double bonds in the fatty acid structures. Therefore, the most significant changes in the content of PUFA were observed among samples stored at room temperature, where various degradation processes, including oxidation, may have played a role. The findings indicate that fatty acids characterized by longer chains and greater degrees of unsaturation are more susceptible to deterioration, potentially due to oxidation [85,86,87].

The decrease in PUFA content and the increase in SFA content for samples stored at room temperature decreased the PUFA/SFA ratio by 23.71% (for samples without access to light) and 31.96% (for samples with access to light). Additionally, there was an increase in the ratio of the total polyunsaturated fatty acids from the n-6/n-3 family by 14.35% (samples without access to light) and 6.82% (samples with access to light).

After 12 months of storing the flour at temperatures of 4 °C and −18 °C, the fatty acid composition of the samples compared to those stored at room temperature was more favorable from a human nutrition perspective. This is because higher a PUFA content and PUFA/SFA ratio were demonstrated, along with a lower SFA content and PUFA n-6/n-3 ratio.

Among the most commonly used indicators for assessing the nutritional value of fat are the PUFA/SFA ratio and the PUFA n-6/n-3 ratio. To inhibit the development of cardiovascular diseases, it is recommended that the values of these indicators be respectively > 0.45 [88] and 4–5:1 [89]. However, it is essential to note that PUFA/SFA is a general indicator and is unsuitable for assessing food’s atherogenicity [63]. This is because specific SFA and PUFA have different metabolic effects [88]. They may either prevent or contribute to the development of cardiovascular diseases (such as atherosclerosis and coronary thrombosis). Therefore, atherogenicity (AI) and thrombogenicity (TI) indices have been developed [63,68], which indicate the direction of the effects of consumed lipids. The atherogenicity index relates to the ratio between saturated fatty acids (pro-atherogenic), which promote lipid deposition in blood vessel walls, and unsaturated fatty acids (anti-atherogenic), which reduce cholesterol levels and protect against coronary artery disease. Meanwhile, the thrombogenicity index indicates the tendency to form clots in blood vessels. It is known that among SFA, the most atherogenic and thrombogenic are the fatty acids C14:0 and C16:0, while C18:0 is considered neutral in terms of atherogenicity but thrombogenic [63]. Unsaturated fatty acids (including MUFA) exhibit anti-atherosclerotic and antithrombotic effects [68,90,91]. The lower the AI and TI values of a product, the higher its nutritional value, and its consumption may reduce the risk of coronary heart disease [63]. Conversely, the higher the hypocholesterolemic to hypercholesterolemic fatty acids ratio, the more suitable the fat is for human nutrition [70].

The significant differences in the fatty acid profiles resulting from different storage conditions led to notable discrepancies in the determined dietary indicators (Table 3). Specifically, the variations in saturated and unsaturated fatty acid levels under different storage conditions directly influenced key nutritional indices. After a 12-month storage period, the most favorable proportion of fatty acids with hypo- and hypercholesterolemic effects, as well as the weakest atherogenic and thrombogenic effects, were observed in samples stored at temperatures of −18 °C and +4 °C. The values of the AI, TI, and h/H indicators for these samples did not differ from the values determined in the control sample. The values of the atherogenicity (AI), thrombogenicity (TI), and h/H indicators were at similar levels, amounting to 0.43 and 0.45; 1.07 and 1.10; and 2.27 and 2.20, respectively, for flour stored at temperatures of −18 °C and +4 °C.

The assessment of the oxidative stability of cricket flour stored at different temperatures (Table 4) showed that 12 months of storage at 20 °C with exposure to light causes significant progress of fat hydrolysis (the highest AV = 6.178 mg KOH/g of fat), which is essential for the acceleration of the oxidation reaction [92]. The lowest acid value (0.15 mg KOH/g of fat) was observed in the control sample (Table 4), indicating the minimal degree of fat decomposition. The results for samples stored for 12 months at 4 °C and −18 °C were similar and lower than those stored at 20 °C (Table 4). Therefore, lower storage temperatures slow hydrolysis reactions, which are crucial to accelerating oxidation.

The lowest peroxide value (0.038 μEq O_2_/g of fat) and anisidine value (2.18) were observed in samples stored at −18 °C (Table 4). The highest values of these two parameters were found in samples stored at 20 °C with light exposure. Samples stored at 4 °C and 20 °C had higher PV values than the control sample (Table 4). On the other hand, anisidine values did not differ significantly between the control sample and those stored at 4 °C and 20 °C. 

The lowest storage temperature also prevented the formation of hydroperoxides in fat from cricket flour, which was demonstrated by the very low peroxide value of the fat. The exposure to light resulted in a significant increase in lipid oxidation in samples stored at room temperature, possibly due to light-induced degradation of compounds present in the flour, such as retinol, with the formation of reactive species [93]. Light and non-reduced temperatures also formed the most significant secondary oxidation products in the fat extracted from the stored flour samples. In contrast, a lack of light and lower storage temperatures for 12 months did not accelerate the propagation of fat oxidation compared to fresh samples. This means that only a free radical chain reaction of oxidation took place in the lipids of these samples. Among the factors inhibiting the formation of secondary oxidation products in fat, the phospholipids could have played a potential role [94].

Accelerated oxidation tests using the DSC method showed the highest oxidative stability of non-stored flour, as indicated by the highest oxidation initiation temperature (225.59 °C). In contrast, higher susceptibility to elevated temperatures (from 50 to 350 °C) and oxygen levels was observed in each storage condition compared to the control (4 °C, 20 °C, 20 °C with light exposure, and −18 °C), with freezing the sample having the least effect (Table 4). These results indicate that the fats in these samples were more prone to oxidation due to the higher content of primary oxidation products involved in chain reactions during the propagation phase. However, the slightly lower oxidative stability of the sample stored at −18 °C could result from the partial degradation of antioxidants in flour during a prolonged period of storage [95]. 

The maximum oxidation temperatures (Tmax), indicating the end of the propagation phase and termination of fat oxidation, were similar across all samples (Table 4). Comparable values of the temperatures at which oxidation termination (Tmax) occurred for all samples stored and for the control sample may indicate the significant antioxidant activity of the Maillard reaction products forming in cricket flour samples at high temperatures [96].

The results of this study are consistent with those of other researchers, who have emphasized the importance of storage temperature for the oxidative stability of cricket flour [60,61,62], indicating that lower storage temperatures delay oxidation processes [60,62] as evidenced by lower acid values, peroxide values [60,61,62], and anisidine values [60], leading to the greater stability of fatty acids, which undergo fewer changes at lower storage temperatures [60].

### Study Limitations

The insect flour was purchased commercially from an online store. Therefore, the conditions in which the insect flour was stored are unknown, which may affect its initial quality. Furthermore, the specific methods used for slaughtering and powdering the crickets and any dehydration, drying, or thermal processing techniques not disclosed on the product label could have directly influenced the flour’s lipid profile and related characteristics. The results apply only to house cricket (*Acheta domesticus*) flour and may not directly apply to other insect flours. Comparative studies with other edible insects may help generalize the results.

## 4. Conclusions

As the spectral data proved consistent with biochemical data on the composition of cricket flour, FTIR/ATR analyses can be used to analyze this food material. Storage temperature had a significant impact on changes occurring in fatty acids and the calculated dietary indicators based on them. Storage at room temperature, especially when exposed to light, negatively impacts the oxidative stability of cricket flour fat. Samples stored at 20 °C had higher acid values (AV), peroxide values (PV), and anisidine values (p-AV) compared to samples stored at lower temperatures (4 °C and −18 °C). 

Storing cricket flour at lower temperatures is crucial to limiting fat degradation. Higher temperatures and the presence of light significantly accelerate oxidation processes, decrease thermal stability, and lead to more significant changes in fat quality. From a human nutrition perspective, it is more favorable to store insect flour in refrigerated conditions, because the nutritional value of the fat does not undergo adverse changes when the flour is refrigerated. 

Based on the results, several recommendations can be formulated to help ensure good fat preservation during the storage of cricket flour. Amber containers or dark packaging materials are advised to prevent light-induced degradation. Additionally, maintaining controlled moisture levels and temperatures during transportation and storage is essential to preserving the quality of the fats in the insect flour. 

## Figures and Tables

**Table 1:** Storage conditions.

Storage Location	Temperature	Access to Light	Humidity [%]
Refrigerator	4 °C	No access	55
Freezer	−18 °C	No access	20
In the laboratory cabinet	20 °C	No access	45
Outside the laboratory Cabinet	20 °C	With access (illumination intensity 500 lx)	45

**Table 2:** Principal Component Analysis results for FTIR band intensities in insect flour stored under different conditions.

Wavenumber (cm^−1^)	PC1 (%)	PC2 (%)	Total Variance (%)
3007	83.46	16.38	99.84
964	83.26	16.40	99.66
716	83.32	16.40	99.72
1745	82.6	16.41	99.01
1736	83.45	16.39	99.84
1171	83.25	16.40	99.65
1237	83.41	16.40	99.81

PC1—principal component 1, PC2—principal component 2.

**Table 3:** Fatty acid content and dietary indicators in insect flour during 12 months of storage in different conditions [%].

Fatty Acids and Dietary Indicators	A (Control Sample)	B (4 °C)	C (20 °C)	D (20 °C + Light)	E (−18 °C)
SFA	36.22 ^a^ ± 1.84	38.40 ^ab^ ± 1.23	40.49 ^bc^ ± 1.06	42.44 ^c^ ± 1.00	37.28 ^a^ ± 1.70
C 12:0					-
C 14:0	0.47 ^a^ ± 0.02	0.62 ^b^ ± 0.00	0.76 ^c^ ± 0.00	1.10 ^d^ ± 0.03	0.45 ^a^ ± 0.02
C 16:0	25.57 ^a^ ± 1.30	25.23 ^a^ ± 0.81	26.81 ^ab^ ± 0.70	27.59 ^b^ ± 0.65	25.03 ^a^ ± 1.13
C 17:0	0.2 ± 0.01				-
C 18:0	8.98 ^a^ ± 0.46	11.15 ^bc^ ± 0.36	11.38 ^bc^ ± 0.30	11.63 ^cd^ ± 0.27	10.70 ^b^ ± 0.48
C 20:0	0.63 ^a^ ± 0.03	0.87 ^b^ ± 0.03	0.84 ^b^ ± 0.02	0.85 ^b^ ± 0.02	0.61 ^a^ ± 0.02
C 22:0	0.2 ± 0.01				0.22 ^b^ ± 0.01
MUFA	28.66 ^a^ ± 1.46	27.86 ^a^ ± 0.80	28.88 ^a^ ± 0.76	29.63 ^a^ ± 0.70	28.23 ^a^ ± 1.30
C 16:1	0.63 ^a^ ± 0.03	0.62 ^a^ ± 0.02	0.69 ^c^ ± 0.02	0.76 ^d^ ± 0.02	0.57 ^b^ ± 0.03
C 18:1	27.63 ^a^ ± 1.25	25.84 ^b^ ± 0.66	27.65 ^a^ ± 0.65	27.76 ^a^ ± 0.59	24.29 ^b^ ± 1.10
C 24:1				0.42 ± 0.01	-
PUFA	35.16 ^a^ ± 1.79	33.70 ^a^ ± 1.17	30.63 ^b^ ± 1.06	27.84 ^c^ ± 0.66	34.50 ^a^ ± 1.55
C 18:2 n-6	33.27 ^a^ ± 1.69	32.32 ^a^ ± 1.11	29.11 ^b^ ± 1.01	26.15 ^c^ ± 0.62	32.82 ^a^ ± 1.50
C 18:3 n-3	1.14 ^a^ ± 0.06	0.75 ^c^ ± 0.02	0.86 ^b^ ± 0.03	0.85 ^b^ ± 0.00	0.87 ^b^ ± 0.03
UFA	63.82 ^a^ ± 3.24	61.56 ^ab^ ± 1.98	59.51 ^ab^ ± 1.82	57.56 ^b^ ± 1.36	62.72 ^a^ ± 2.85
PUFA/SFA	0.97 ^a^ ± 0.00	0.91 ^b^ ± 0.00	0.74 ^d^ ± 0.02	0.66 ^e^ ± 0.00	0.92 ^b^ ± 0.00
PUFA n-3	1.38 ^a^ ± 0.07	1.11 ^b^ ± 0.04	1.07 ^b^ ± 0.03	1.02 ^b^ ± 0.02	1.10 ^b^ ± 0.05
PUFA n-6	33.30 ^a^ ± 1.69	32.40 ^a^ ± 1.12	29.56 ^b^ ± 1.01	26.32 ^c^ ± 0.62	32.91 ^a^ ± 1.50
PUFA n-6/PUFA n-3	24.18 ^a^ ± 0.00	29.64 ^b^ ± 0.05	27.65 ^c^ ± 0.17	25.83 ^d^ ± 0.10	29.92 ^b^ ± 0.00
AI	0.43 ^a^ ± 0.00	0.45 ^a^ ± 0.00	0.50 ^c^ ± 0.00	0.56 ^d^ ± 0.00	0.43 ^a^ ± 0.00
TI	1.00 ^a^ ± 0.00	1.10 ^b^ ± 0.00	1.20 ^c^ ± 0.00	1.30 ^d^ ± 0.00	1.07 ^b^ ± 0.00
h/H	2.38 ^a^ ± 0.01	2.20 ^b^ ± 0.00	2.09 ^c^ ± 0.01	1.91 ^d^ ± 0.00	2.27 ^b^ ± 0.00

Means with different letters in the same row differ at *p* ˂ 0.05. SFA—saturated fatty acid, MUFA—monounsaturated fatty acids, PUFA—polyunsaturated fatty acid, UFA—unsaturated fatty acid, AI—atherogenic index, TI—thrombogenic index, h/H—hypocholesterolemic/hypercholesterolemic ratio.

**Table 4:** Impact of Different Storage Conditions on Fat Oxidation and Stability Indicators.

Indicators	A (Control Sample)	B (4 °C)	C (20 °C)	D (20 °C + Light)	E (−18 °C)
AV (mg KOH/g of fat)	0.15 ^a^ ± 0.00	0.611 ^a^ ± 0.00	1.704 ^b^ ± 0.22	6.178 ^c^ ± 0.41	0.596 ^a^ ± 0.04
PV (μEq O_2_/g of fat)	0.82 ^a^ ± 0.08	3.20 ^b^ ± 0.33	3.71 ^b^ ± 0.36	6.03 ^c^ ± 0.27	0.038 ^d^ ± 0.02
*p*-AV	2.53 ^a^ ± 0.14	2.23 ^a^ ± 0.16	2.35 ^a^ ± 0.12	6.63 ^b^ ± 0.02	2.18 ^a^ ± 0.16
DSC T_on_ (°C)	225.59 ^a^ ± 8.59	188.91 ^b^ ± 0.61	194.45 ^b^ ± 4.27	185.56 ^b^ ± 0.63	212.46 ^c^ ± 3.04
DSC T_max_ (°C)	319.03 ± 1.18	318.17 ± 1.89	317.15 ± 2.21	321.26 ± 0.18	318.78 ± 0.34

Means with different letters in the same row differ at *p* ˂ 0.05. AV—acid value, PV—peroxide value, *p*-AV—para anisidin value, DSC T_on_—temperature of the oxidation induction measured by DSC, DSC T_max_—temperature of the oxidation termination measured by DSC.

## Data Availability

The original contributions presented in the study are included in the article, further inquiries can be directed to the corresponding author.
